# Late-Onset Invasive Aspergillosis Following Acalabrutinib in 2 Patients With Chronic Lymphocytic Leukemia: A Case Report

**DOI:** 10.1093/ofid/ofag683

**Published:** 2026-10-23

**Authors:** Hugo Bes-Berlandier, Bochra Sedaki, Dikélélé Elessa, Pilar Dutari, Anne Calleja, Barbara Foucher, Jazil Hassam, Hassina Guerchouche, Stephane Hernandes, Pierre-Christophe Leoni, Pierre Berger, Ferdinand Willetard, David Delarbre, Philippe Lavrard, Gabriel Brisou

**Affiliations:** Infectiologie Transversale, Institut Paoli Calmettes, Marseille, France; Département d'hématologie, Institut Paoli Calmettes, Marseille, France; Département d'hématologie, Institut Paoli Calmettes, Marseille, France; Département d'hématologie, Institut Paoli Calmettes, Marseille, France; Département d'hématologie, Institut Paoli Calmettes, Marseille, France; Department of Biology, Military Teaching Hospital Sainte-Anne, Toulon, France; Department is Service de medecine interne, Service de Médecine Interne, Corte, France; Department is Service de medecine interne, Service de Médecine Interne, Corte, France; Department is Service de medecine interne, Service de Médecine Interne, Corte, France; Department is Service de medecine interne, Service de Médecine Interne, Corte, France; Infectiologie Transversale, Institut Paoli Calmettes, Marseille, France; Département d'hématologie, Institut Paoli Calmettes, Marseille, France; Division of Internal Medicine and Infectious Diseases, Military Teaching Hospital Sainte-Anne, Toulon, France; Division of Internal Medicine and Infectious Diseases, Military Teaching Hospital Sainte-Anne, Toulon, France; Département d'hématologie, Institut Paoli Calmettes, Marseille, France

**Keywords:** acalabrutinib, aspergillosis, Bruton tyrosine kinase inhibitors, chronic lymphocytic leukemia

## Abstract

Bruton tyrosine kinase inhibitors (BTKi) have emerged over the past several years as a cornerstone of treatment for chronic lymphocytic leukemia (CLL). While ibrutinib, a first-generation BTKi, was the first in this therapeutic class associated invasive fungal infections (IFI), recent data also reported an increased risk with acalabrutinib, a second-generation BTKi. Acalabrutinib-associated aspergillosis tends to occur relatively early, often within the first few months after BTKi initiation. We describe 2 late-onset cases in CCL occurring 22 months after exposure. One patient had proven aspergillosis with brain abscess and pneumonia, while the other had probable aspergillosis with brain and lung involvement. The 2 patients discontinued BTKi and only one survived. These observations highlight the sustained IFI risk associated with prolonged BTKi therapy. Finally, these findings support a strategy combining vigilant clinical monitoring with regular serum galactomannan assessment in patients with risk factors such as refractory CLL, neutropenia, or structural lung diseases.

Bruton tyrosine kinase inhibitors (BTKi) have significantly transformed the management of chronic lymphocytic leukemia (CLL), offering improved overall survival [1]. Acalabrutinib is a second-generation BTKi with a shorter plasma half-life and greater selectivity for BTK than the first-generation BTKi ibrutinib [1]. The BTK receptor is involved in the TLR9–BTK–calcineurin–NF-κB cascade, which is essential in B-cell development but also in macrophage and neutrophil antifungal responses. Consequently, BTK inhibition by these therapies compromises innate and adaptive immunity, increasing susceptibility to invasive fungal infections (IFI) [2]. In ibrutinib-treated patients, several authors have reported IFI, particularly cerebral aspergillosis, with mortality rates up to 51% [3]. However, the prevalence of IFIs in those treated with second-generation BTKi remained unclear. Thus, Srisurapanont et al conducted a systematic review and meta-analysis involving 23 737 patients, which revealed no significant difference in the pooled prevalence of fungal infections between ibrutinib (1.75%) and acalabrutinib (1.31%) [4]. Therefore, another review and meta-analysis found that aspergillosis during acalabrutinib therapy occurred preferentially within the first months of the therapy [5]. We report 2 cases of late-onset aspergillosis in patients with CLL treated with acalabrutinib.

## PATIENT 1

A 73-year-old man with significant comorbidities, including diabetes and chronic kidney disease, was diagnosed with CLL in 2008. First-line therapy consisted of fludarabine, cyclophosphamide, and rituximab (FCR), achieving complete remission with minimal residual disease positivity, followed by inclusion in the RESIDIUM study and lenalidomide consolidation in 2018 as second-line treatment (see Table 1). After several years of remission, the patient relapsed in 2023 with massive bone marrow infiltration and pancytopenia. In April 2023, he developed COVID-19-associated right pulmonary aspergillosis and was treated with itraconazole for 3 months, resulting in complete clinical, microbiological, and radiological response. In May 2023, acalabrutinib was initiated as later-line therapy, leading to hematologic complete response but persistent grade 3 toxic neutropenia requiring growth factor support. The patient was also receiving polyvalent immunoglobulin supplementation for hypogammaglobulinemia.

**Table 1.** Characteristics and Outcomes of the 2 Patients With Acalabrutinib-Associated Aspergillosis

	Gender	Comorbidities and Past Infections	Hematologic Disease	Prior Hematologic Therapies	Time on ACALA (m)^a^	Neutropenia at the Time of IFI Diagnosis	EORTC Classification of IFI	Sites of Infection	Management of ACALA	Antifungal Treatment	Outcome
Patient 1 73 yo	Male	Diabetes and chronic kidney disease, IPA in 2023	Stage 3 CLL with a hematologic complete response	First line: fludarabine, CTX, RTX Second line: lenalidomide consolidation	22	Yes	Probable	Brain and lung	Cessation	VOR then ISA	Alive
Patient 2 74 yo	Female	COPD with bronchiectasis	Stage 3 CLL with a hematologic complete response	First line: venetoclax and OBT	22	No	Proven	Brain and lung	Cessation	VOR	Death in relation with CLL progression

Abbreviations: IFI, invasive fungal infection; ACALA, acalabrutinib; VOR, voriconazole; ISA, isavuconazole; CLL, chronic lymphocytic leukemia; CTX, cyclophosphamide; RTX, rituximab; OBT, obinutuzumab; COPD, chronic obstructive pulmonary disease; IPA, invasive pulmonary aspergillosis; yo, years old.

^a^At the time of IFI diagnosis.

In March 2025, he was admitted to the hospital with cough, fever, and neutropenia (1.4 G/L). Immunoglobulin levels were normal. Chest Computed Tomography (CT) revealed an 8 cm cavitary consolidation (Figure 1*A*) in the left upper lobe and a 2.7 cm cavitary nodule in the left lower lobe.

**Figure 1.**
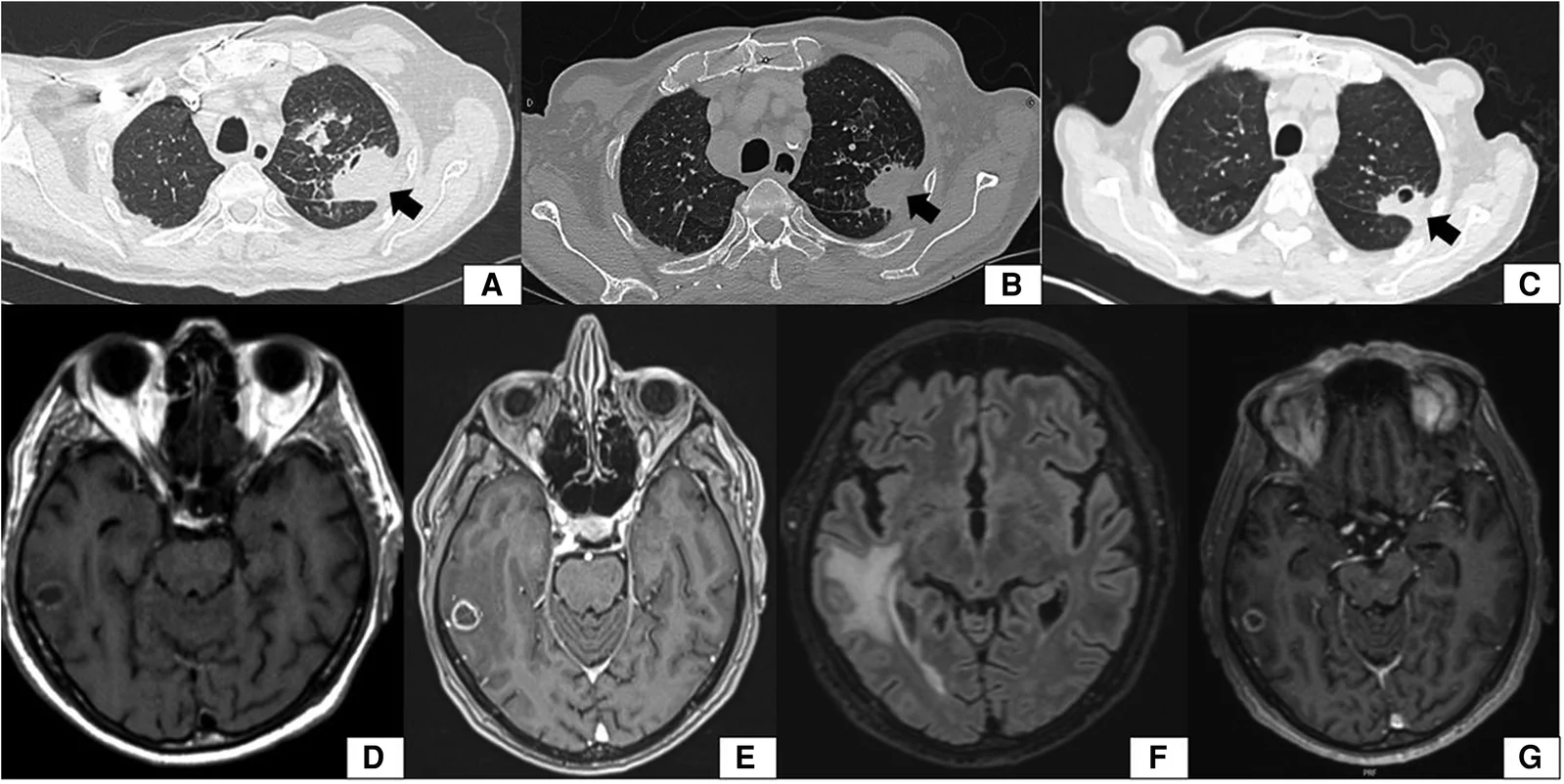
Radiological evolution of lung involvement in patient 1 at diagnosis (*A*), at 2 months (*B*), and at 5 months of antifungal treatment (*C*). Radiological evolution of brain involvement in patient 1 at diagnosis (*D*), at 2 months (*E* and *F*), and at 7 months of antifungal treatment initiation (*G*). In March 2025, chest CT revealed an 8 cm cavitary consolidation (thick black arrow) in the left upper lobe (*A*). In May 2025, chest CT demonstrated significant regression of the cavitary consolidation (thick black arrow, *B*). In August 2025, chest CT showed continued improvement of the cavitary consolidation (thick black arrow, *C*). In March 2025, T1-weighted MRI revealed 2 ring-enhancing lesions, each 1.5 cm in size, located in the right temporal region and the left cerebellar hemisphere (*D*). In May 2025, 3D T1-weighted MRI with gadolinium contrast demonstrated increased enhancement of the right temporal lesion (*E*) along with worsening perilesional edema (*F*). In June 2025, T1-weighted MRI with gadolinium contrast showed improvement of the right temporal lesion (*G*).

*Aspergillus fumigatus* had been isolated from bronchoalveolar lavage (BAL) in March 2025. Unfortunately, no antifungal susceptibility testing was performed. Serum galactomannan antigen was negative. Given the diagnosis of probable invasive pulmonary aspergillosis, voriconazole was initiated and acalabrutinib was discontinued.

Brain Magnetic Resonance Imaging (MRI) was performed to assess for other fungal localizations and showed 2 ring-enhancing lesions, each 1.5 cm in size, located in the right temporal region and the left cerebellar hemisphere (1D). These MRI findings, together with the BAL results, were consistent with cerebral aspergillosis.

In May 2025, brain MRI demonstrated increased contrast enhancement of the right temporal lesion (Figure 1*E*), along with worsening perilesional edema (Figure 1*F*) while chest CT scan showed a decrease in the cavitary consolidation (Figure 1*B*). Given the absence of clinical neurological deterioration, we decided to continue voriconazole. Follow-up MRI in June 2025 revealed a significant decrease in the right temporal lesion (Figure 1*G*) and in the cerebellar lesion. The voriconazole trough level was 1.7 mg/L, prompting a dosage increase to achieve a target above 2 mg/L and ensure therapeutic voriconazole levels in the cerebrospinal fluid [6].

In August 2025, MRI and chest CT scan (Figure 1*C*) confirmed persistent improvement. Following venetoclax initiation, voriconazole was switched to isavuconazole to minimize pharmacologic interaction. Venetoclax dosing was subsequently reduced due to isavuconazole's moderate CYP3A4 inhibition [7]. As of June 2026, he was still alive but had persistent neutropenia.

## PATIENT 2

A 74-year-old woman with a history of chronic obstructive pulmonary disease COPD with bronchiectasis, a well-established independent risk factor for pulmonary aspergillosis, was admitted in March 2026 for altered consciousness and mutism. She also had a stage 3 CLL in hematologic complete response on second-generation BTKi. She had been receiving acalabrutinib since May 2024, following first-line treatment with venetoclax and obinutuzumab, which had been discontinued 1 month after initiation because of poor tolerance. The patient's immunoglobulin levels were normal.

She was afebrile and had neutrophil and lymphocyte counts of 2.3 G/L and 0.7 G/L, respectively.

Brain MRI revealed a 3 cm right frontal mass with central contrast enhancement, as well as a 12 mm rightward subfalcine herniation. Given the initial suspicion of a brain tumor, a complete exeresis of the mass was performed, and empirical antibiotic therapy with meropenem and linezolid was initiated postoperatively. However, *A fumigatus* was isolated on the mycologic culture of the surgical specimen, confirming the diagnosis of proven brain aspergillosis. This led to initiation of voriconazole and discontinuation of antibiotics. Chest CT revealed tree-in-bud micronodules in the right lung associated with peribronchial consolidations, suggestive of invasive bronchopulmonary aspergillosis. Serum galactomannan antigen was negative. Given the disseminated aspergillosis, acalabrutinib was discontinued. The patient demonstrated neurological and radiological improvement but died from CLL progression in June 2026.

## DISCUSSION

We report 2 cases of late-onset aspergillosis in patients with CLL treated with acalabrutinib. The delayed onset of fungal infection in our patients (22 months after BTKi initiation for each patient) contrasts with the literature. Indeed, a recent review including 7 patients with aspergillosis found a mean delay of 2.2 months between acalabrutinib initiation and fungal infection [5]. The early onset of aspergillosis may align with Blaize et al's observations of impaired oxidative burst and neutrophil killing capacity in patients with acalabrutinib-associated aspergillosis as early as 1 month after BTKi initiation [8].

Management of CLL treatment remains challenging in the setting of aspergillosis. Acalabrutinib discontinuation in our 2 patients led to improvement of the fungal infection but was also associated with fatal CLL progression in patient 2. Gibert et al conducted a systematic review of 211 patients with CLL lymphoma who were treated for invasive mold infections (including 50% aspergillosis) following to first- and second-generation BTKi. They reported that BTKi discontinuation was associated with improved survival compared to continuation (72.9% vs 45.5%) but did not perform subgroup analysis in patients with aspergillosis [5]. Furthermore, several experts advocate discontinuing ibrutinib in grade 4 infections until the infection is adequately treated and improves to at least grade 3 [9].

Moreover, antifungal prophylaxis is not routinely recommended by ECIL guidelines for CLL patients receiving BTKi; however, it may be considered in selected patients with refractory disease treated with BTKi or those with prolonged neutropenia [10]. More recently, a meta-analysis found that at least 3 previous lines of therapy and corticosteroid use were independent risk factors of IFI in patient receiving BTKi. In contrast, these findings do not align with the profiles of our patients, who had undergone only 2 and 1 prior lines of therapy, respectively. Additionally, patient 2 developed aspergillosis without any of the risk factors described by ECIL guidelines, underscoring the significant role of COPD with bronchiectasis in this fungal infection. Thus, structural lung diseases should be taken into account in the individualized risk assessment of patients with CLL treated with BTKi. Furthermore, the high risk of IFI in patient 1 (due to prior aspergillosis and prolonged neutropenia) could have prompted a discussion about antifungal prophylaxis with posaconazole after BTKi initiation. However, since posaconazole is a strong CYP3A4 inhibitor and acalabrutinib is a CYP3A4 substrate, a dose reduction of second-generation BTKi to 100 mg once daily—per FDA labeling—is required [7]. Therefore, the significant drug–drug interactions and pooled prevalence of invasive IFI in patients receiving BTKi for CLL (eg, 2.4% in a systematic review and meta-analysis) emphasize the need of implementing a surveillance-based strategy in high-risk populations, rather than routinely administering antifungal prophylaxis [4]. Such monitoring should be adapted to an outpatient setting and integrate regular clinical follow-up with serum galactomannan antigen. However, our 2 patients had negative galactomannan antigen, highlighting the necessity of systematically performing imaging in cases of suspicion of aspergillosis.

We acknowledge several limitations. First, the multiple additional risk factors of IFI present in each case (eg, prior therapies, prior aspergillosis, neutropenia, COPD) warrant caution when attributing aspergillosis occurrence solely to acalabrutinib. Indeed, all these factors might have contributed to cumulative immunosuppression, increasing susceptibility to IFI.

Nevertheless, none of our patients had received prolonged corticosteroids in the period between acalabrutinib initiation and IFI diagnosis. Second, the first episode of aspergillosis described in patient 1—which occurred before starting acalabrutinib—raises the question of whether this was a relapse of IFI rather than a new episode. However, the different localizations of pulmonary aspergillosis in 2023 and 2025 support the hypothesis of a new episode, as relapses typically involve the same site as the initial infection [6]. Finally, the median onset of 2.2 months described in the review by Gibert et al may not be representative due to the low number of acalabrutinib-associated aspergillosis cases [5].

In conclusion, these cases highlight the persistent long-term risk of aspergillosis in patients receiving acalabrutinib. These findings also support the need for vigilant clinical monitoring with regular assessment of serum galactomannan antigen in patients with risk factors such as refractory CLL, neutropenia, or structural lung diseases.
